# Education Research: Comparison of Lecture-Based and Direct Instruction Didactics to Improve Neurologic Examination Confidence in Neurology Clerkship Students

**DOI:** 10.1212/NE9.0000000000200236

**Published:** 2025-08-26

**Authors:** Jonathan Trout, Matthew H. Roberts, Deborah Carver, Paola Martinez

**Affiliations:** 1 Department of Neurology, University of Utah, Salt Lake City;; 2 School of Medicine Greenville, University of South Carolina;; 3 Department of Neurology, Prisma Health Upstate, Greenville, SC; and; 4 Department of Neurology, University of Texas Health San Antonio.

## Abstract

**Background and Objectives:**

Teaching medical students how to perform an accurate neurologic examination (NE) is a primary objective of the neurology clerkship. Despite this emphasis, medical students often lack confidence in performing the NE. There is limited research on the effect of teacher-centered instruction methods on NE confidence. We designed this study to compare the impact of a lecture-based (LB) didactic and a direct instruction (DI) didactic on medical students' NE confidence during the neurology clerkship.

**Methods:**

We conducted a teacher-centered, NE didactic during a four-week neurology clerkship. Third-year medical students (MS3s) were assigned to an LB didactic providing an overview of the NE or a DI didactic focused on examination pitfalls and practice opportunities. Preclerkship and postclerkship surveys assessed self-reported confidence of MS3s in performing various aspects of the NE and the perceived benefit of each didactic on examination skills. Clerkship evaluations of physical examination skills assessed the impact of each teaching method on NE performance. The primary outcome was change in composite NE confidence scores from baseline. Secondary outcomes were change in individual NE confidence scores from baseline, perceived didactic benefit, and clerkship physical examination scores.

**Results:**

A total of 103 MS3s participated in our study (LB = 52, DI = 51). Change in NE confidence scores from baseline did not differ between groups (LB mean = 7.62, DI mean = 7.66, *p* = 0.97, *d* = 0.007). Presurvey strength testing confidence scores were higher in the DI group (LB mean = 3.04, DI mean = 3.44, *p* = 0.038, *d* = 0.46). There were no other significant differences in other confidence scores between groups (*p* > 0.13). Students reported higher agreement with the DI didactic being beneficial for their NE skills than the LB didactic (LB mean = 3.97, DI mean = 4.52, *p* = 0.0013, *d* = 0.82). Clerkship physical examination evaluation scores did not differ between groups (LB and DI median = 4, interquartile range [IQR] = 3–4, *p* = 0.074, *r* = 0.070).

**Discussion:**

Emphasizing challenging NE pitfalls with DI is perceived by students to be more beneficial for learning examination skills than LB methods. However, DI is not clearly superior to LB teaching methods in improving NE confidence. Future research could further evaluate the efficacy of DI teaching methods with randomized trials and interval assessment of their effects on NE confidence throughout the neurology clerkship.

## Introduction

Teaching medical students how to perform an accurate and reliable neurologic examination (NE) is a primary objective of medical undergraduate education and, more specifically, the neurology clerkship.^1-4^ Despite students understanding which portions of the NE are most important, medical students often do not feel confident performing the NE or examining patients presenting with relevant concerns.^5^ In addition, students are reported to have more difficulty with some portions of the examination than others, such as deep tendon reflex and strength testing.^6^ Given the increasing incidence and prevalence of neurologic disorders, it is essential to create engaging educational materials and interventions that improve medical students' confidence in their ability to perform an effective NE, regardless of the specialty they ultimately pursue.^7^

Recognizing the importance of teaching the NE to medical students, educators have investigated various methods for improving NE teaching. Several studies on teaching the NE focus on singular aspects of the examination, such as reflexes^8,9^ and peripheral nerves,^10^ and have noted significant improvements in these specific skills. A variety of other methods have been used to improve NE skills, such as students-as-teachers and preclinical mock rounds,^11^ standardized patient sessions,^12^ augmented/virtual reality simulation,^13^ immediate bedside feedback,^14^ virtual lectures,^15^ blended learning/flipped classroom,^16^ and problem-based learning strategies.^17^ Educators also frequently use traditional, teacher-centered classroom methods to teach neurologic clinical reasoning and examination skills. Teacher-centered curricula are those that focus on instructors delivering information without much or any student engagement.^18^ Surprisingly, however, there is no literature on how teacher-centered learning methods affect medical students' confidence in performing a variety of NE skills.

To address this gap in the literature, we developed and implemented a novel teacher-centered, direct instruction (DI) didactic into the neurology clerkship that was designed to increase confidence of third-year medical students (MS3s) in various aspects of the NE. We hypothesized that this innovative, pitfall-oriented didactic approach, paired with hands-on practice, would lead to increased confidence in performing the NE by medical students. We also hypothesized that this novel DI didactic would result in improved student perceived didactic benefit and clerkship NE performance.

## Methods

### Curriculum Development and Content

We developed and distributed 2 qualitative surveys (one to MS3s and fourth-year medical students and another to residents, fellows, and faculty (RFF) at our institution) asking about areas of the NE they perceived as most difficult for students. For both surveys, we divided the NE into 6 parts (i.e., cranial nerves, motor, sensory, reflexes, coordination, and gait). We asked respondents to identify 2 specific areas of the NE that students find most challenging to perform. In addition, respondents were asked to describe, for each of the 6 parts of the NE, which aspects are most difficult for learners. RFF members also shared valuable “tips and tricks” they use to help students enhance their examination skills.

To prevent crossover of materials between groups, MS3s in their neurology clerkship were assigned, based on academic calendar block, to receive either a lecture-based (LB; blocks 1–3) or DI (blocks 4–6) didactic. The control, teacher-centered, LB didactic for our clerkship students consisted of an overview of each major aspect of the NE (including mental status) without opportunities to practice examination skills. This session was taught by our neurology clerkship directors and selected as a control because of it already being in the clerkship orientation curriculum for several years.

The teacher-centered, DI didactic was developed by neurology clerkship faculty and a fourth-year medical student (MS4). The session was taught by an MS4 pursuing neurology residency and replaced the traditional didactic in the neurology clerkship orientation curriculum. Recognizing the complexity of the NE and how overwhelming it can be for learners, we designed the DI didactic to focus on and emphasize common NE pitfalls. These pitfalls were inspired by comments found in the qualitative surveys and integrated into the DI didactic in the form of 9 memorable tips referred to as “don'ts” (Table 1). Each item provides a brief “don't” to highlight common mistakes students make while performing the NE. These points were designed to help students recognize and avoid frequent errors, ensuring a more accurate and efficient assessment of neurologic function. For example, “don't give their eyes whiplash” was a reminder for students to slowly test eye movements for subtle findings, such as smooth pursuit, cranial nerve palsies, and nystagmus, which may otherwise be missed by moving quickly through the examination. After verbal explanation of each pitfall and demonstration of how to accurately perform each related NE skill by the teacher and a facilitator, students were provided time to engage in observed, paired practice of each skill. Skills covered included visual fields, extraocular movements, strength, reflexes, and coordination testing. Owing to time constraints, the “don'ts” for sensory and gait testing were only verbal discussions and visual demonstrations.

**Table 1:** List of 9 “Don'ts” Used in the Direct Instruction Didactic Aimed at Helping Students Recognize and Learn From Frequent Neurologic Examination Pitfalls

Related neurologic examination skill	“Don'ts”
Visual fields	Don't wiggle your fingers
Extraocular movements	Don't give their eyes whiplash
Strength	Don't test all muscles at once
	Don't miss subtle weakness
Sensory	Don't keep the sensory exam subjective
Reflexes	Don't use a stethoscope as a hammer
	Don't forget to distract your patient
Coordination	Don't forget to fully extend their arms
Gait	Don't keep them in the bed

Recognizing the importance of individual practice and feedback,^19,20^ we organized the session to provide practice and feedback opportunities to solidify proper examination technique. These aspects of the DI didactic were inspired by instrumental adult learning theories,^21^ namely cognitive load theory, a theory that emphasizes the benefits of presenting novel information in limited amounts to promote learning,^22^ and behaviorist theory, which postulates the importance of feedback and reinforcement in learning new skills.^23^ To provide feedback to learners, the DI didactic also had multiple facilitators for each session, including neurology clerkship directors, neurology residents, and MS4s applying to neurology residency. The novel didactic also incorporated various other “tips and tricks” provided in the qualitative surveys from RFF and allowed time for questions from students.

### Curriculum Evaluation

To evaluate our intervention, presurveys and postsurveys were distributed to students before orientation on the first day of the clerkship and 3–4 weeks after each session, respectively. All participants were University of Texas Health San Antonio (UTHSA) MS3s participating in their clinical neurology clerkship during the 2023–2024 academic year. Presurveys and postsurveys assessed students' confidence in performing some of the more commonly used NE skills, namely assessing visual fields; extraocular movements; strength testing; and brachioradialis, triceps, and ankle deep tendon reflexes (Kirkpatrick level 2). Each of the individual aspects of the NE was combined into a composite NE confidence score (range 0–40). The change in composite NE confidence scores from baseline (difference between presurvey and postsurvey measurements) served as the primary outcome of this study.

Postsurveys also contained a question evaluating the perceived benefit of each didactic on their NE skills (Kirkpatrick level 2). Each question was scored on a 1–5 Likert scale (“strongly disagree,” “disagree,” “neutral,” “agree,” and “strongly agree”). Data were also extracted from clinical evaluations of students from residents and faculty to evaluate the impact of the DI didactic on physical examination performance during the clerkship (Kirkpatrick level 3). These scores were also rated on a 1–5 Likert scale (“Often demonstrates incorrect technique or performs inappropriate exam; misses major findings,” “Sometimes demonstrates incorrect technique or performs inappropriate exam; misses major findings,” “Exam is generally appropriate in scope and technique; identifies major pertinent findings,” “Exam is frequently thorough, focused, efficient, using appropriate technique; identifies major findings and occasionally subtle findings independently,” and “Exam is consistently thorough and accurate even on complicated patients; independently identifies and interprets key and subtle findings; uses advanced technique when appropriate”). Change in individual NE aspect confidence from baseline, perceived benefit on NE skills, and clerkship physical examination evaluations served as secondary outcomes for this study.

### Data Analysis

An a priori power analysis was performed using G*Power version 3.1.9.7 with an effect size of 0.80, significance criterion of α of 0.05, and power of 0.95.^24^ These values were selected because of a lack of previous studies reporting student NE confidence scores that could be used as references for our power analysis. With these criteria, the minimum total sample size needed to detect a significant difference in the primary outcome between groups using *t* tests was *n* = 84 (or 42 per group). Jarque-Bera tests were used to determine normality of each group. Two-tailed, 2-sample equal variance *t* tests were used to compare mean pre-post didactic confidence scores within and between the LB and DI groups. Mann-Whitney *U* tests were used to compare median clerkship NE evaluation scores between the LB and DI groups. Cohen *d* was used to determine the effect size of confidence score analysis. Pearson *r* was used to determine the effect size of clerkship examination evaluation score comparisons. Statistical significance was determined by a *p* value <0.05.

### Standard Protocol Approvals, Registrations, and Patient Consents

Our study was reviewed by the Institutional Review Board at UTHSA and deemed to be exempt because of our study being conducted in established or commonly accepted educational settings and only including interactions involving educational tests and survey procedures.

### Data Availability

Anonymous data that have not been published in this article are available at the request of other investigators. Qualified investigators interested in obtaining access to our data may contact the corresponding author (J.T.).

## Results

### Qualitative Curricular Development Surveys

The descriptive findings from the qualitative curricular development surveys are provided in Table 2. The comments in these surveys demonstrated that students often have difficulty with evaluating many aspects of the NE, including visual fields, extraocular movements, motor strength, deep tendon reflexes, sensation, finger-to-nose testing, and gait. Medical students (*n* = 19) chose up to 2 aspects of the NE they found most difficult to perform. From most to least frequently reported, students indicated difficulty assessing reflexes (percent of respondents: 68%), gait (26%), cranial nerves (21%), strength (16%), sensation (16%), and coordination (11%). RFF (*n* = 10) also chose 2 aspects of the NE they noted students struggled with the most. From most to least frequently reported, they observed that students struggled with testing reflexes (80%), strength (50%), cranial nerves (30%), sensation (20%), coordination (20%), and gait (10%).

**Table 2:** Example Comments From Students, Residents, Fellows, and Faculty (RFF) in the Curricular Development Qualitative Surveys Highlighting the Most Challenging Aspects of the Neurologic Examination for Students

Aspect of the neurologic examination	Challenging aspects of neurologic examination for students, reported by students	Challenging aspects of neurologic examination for students, reported by RFF	Tips to improve neurologic examination
Cranial nerves	1. “Remembering to do all 12”	1. “Visual fields”	1. “Check one eye at a time”
	2. “Visual fields”	2. “Extraocular movements (EOM)”	2. “Asking how many fingers, not if fingers are moving”
	3. “EOM”		3. “Test smooth pursuit”
	4. “Rinne and Weber”		
Motor	1. “Grading strength”	1. “Don't isolate each movement”	1. “Always isolate above and below every joint”
	2. “Isolating movements”	2. “Doing both sides at same time”	2. “Careful positioning”
	3. “Doing them on patient who's in bed”	3. “Differentiating between 4/5 and 5/5”	
Sensory	1. “Remembering correct areas to test pain vs temperature vs light touch”	1. “Do not test all modalities”	1. “Check one large fiber and one small fiber” modality
		2. “Don't test distal to proximal for neuropathy”	2. “Start distally and ascend to assess for” neuropathy
		3. “Doing it too quickly”	
Reflexes	1. “Grading reflexes”	1. “Incorrect technique” and “positioning”	1. “Use an adequate hammer”
	2. “Eliciting reflexes”	2. “Making sure patient is relaxed”	2. “Practice whenever you can”
	3. “Reflexes in someone in a hospital bed”	3. “Using an inadequate hammer”	3. “Distract the patient”
		4. “Don't apply enough strength”	4. “Make sure patient is not flexing muscle you are testing”
Coordination	1. “Remembering to do it”	1. “Not fully extending in finger to nose”	1. “Fully extend arm”
	2. “Finger to nose”	2. “Check both upper and lower limb”	2. “Have patient carry out slow movements during targeting”
	3. “Heel to shin”		
Gait	1. “Describing different types of gait”	1. “Often not done”	1. “Do it as long as it’s safe”
	2. “Remembering to do it all”	2. “Not watching patient in hallway”	2. “Watch patient as they walk into exam room”
			3. “Walk a sufficient distance”

Also included are tips shared by RFF in the qualitative surveys to help students improve their examination skills.

### Evaluation of Didactic Surveys

Tables 3–5 present the findings of the presurveys and postsurveys assessing NE confidence in the LB and DI groups. A total of 103 MS3s participated in our study (LB: *n* = 52; DI: *n* = 51). Completion rates of the presurvey (LB = 49/52; DI = 39/51) and postsurvey (LB = 35/52; DI = 33/51) in each group were high (≥60%). Individual aspect and composite NE confidence scores significantly increased after the clerkship in both groups (*p* < 0.03). Presurvey strength testing confidence scores were significantly higher in the DI group (mean = 3.44, SD = 0.82) than in the LB group (mean = 3.04, SD = 0.91; *p* = 0.038, *d* = 0.46). Other presurvey and postsurvey confidence scores for assessing visual fields, extraocular movements, strength rating, and reflexes did not significantly differ between the LB and DI groups (*p* > 0.05). The presurvey and postsurvey composite NE confidence scores also did not differ between the LB (postsurvey mean = 31.3, SD = 4.73) and DI groups (postsurvey mean = 32.8, SD = 4.91; *p* = 0.20, *d* = 0.35). Change in confidence scores from baseline for each individual NE aspect and the composite NE was not significantly different between groups (*p* > 0.05). Students reported significantly higher agreement with the DI didactic (DI mean = 4.52, SD = 0.57) being helpful for their NE skills than the traditional didactic (LB mean = 3.97, SD = 0.75; *p* = 0.0013, *d* = 0.82).

**Table 3:** Comparison of **Presurvey** Neurologic Examination Confidence Scores Between the Lecture-Based (LB) and Direct Instruction (DI) Didactic Groups Based on Individual Aspects of the Examination

Aspect of the neurologic examination	Pre-LB (*n* = 49/52)	Pre-DI (*n* = 39/51)	*p* value	Cohen *d*
Visual fields	3.18 (0.81)	3.31 (0.86)	0.49	0.15
Oculomotor movement	3.80 (0.82)	3.95 (0.76)	0.37	0.19
Grading strength	2.39 (0.89)	2.69 (1.00)	0.13	0.32
Motor strength	3.04 (0.91)	3.44 (0.82)	0.038^a^	0.46
Grading reflexes	2.27 (0.76)	2.59 (0.82)	0.057	0.41
Triceps reflex	2.94 (0.92)	2.87 (1.00)	0.75	0.069
Brachioradialis reflex	2.78 (0.92)	2.79 (0.95)	0.92	0.021
Ankle reflex	3.31 (0.89)	3.51 (0.94)	0.30	0.22
Composite neurologic examination confidence	23.7 (4.08)	25.2 (4.87)	0.13	0.32

Note: Mean (SD). *p* Value calculated using 2-tailed, 2-sample equal variance *t* tests.

a *p* < 0.05

**Table 4:** Comparison of **Postsurvey** Neurologic Examination Confidence Scores Between the Lecture-Based (LB) and Direct Instruction (DI) Didactic Groups

Aspect of the neurologic examination	Post-LB (*n* = 35/52)	Post-DI (*n* = 33/51)	*p* Value	Cohen *d*
Visual fields	3.91 (0.85)	4.15 (0.76)	0.23	0.29
Oculomotor movement	4.40 (0.65)	4.58 (0.66)	0.27	0.27
Grading strength	4.00 (0.77)	4.18 (0.68)	0.31	0.25
Motor strength	4.14 (0.81)	4.39 (0.61)	0.16	0.35
Grading reflexes	3.54 (0.92)	3.67 (0.99)	0.59	0.13
Triceps reflex	3.63 (0.94)	3.85 (0.97)	0.35	0.23
Brachioradialis reflex	3.80 (0.87)	3.97 (1.07)	0.48	0.17
Ankle reflex	3.89 (0.83)	4.03 (0.92)	0.50	0.17
Composite neurologic examination confidence	31.3 (4.73)	32.8 (4.91)	0.20	0.35

Note: Mean (SD). *p* value calculated using 2-tailed, 2-sample equal variance *t* tests.

**Table 5:** Comparison of Change From Baseline (CFB) in Individual Neurologic Examination Aspects and Composite Neurologic Examination Confidence Scores Between the Lecture-Based (LB) and Direct Instruction (DI) Didactic Groups

Aspect of the neurologic examination	LB CFB	DI CFB	*p* Value	Cohen *d*
Visual fields	0.73 (1.07)	0.84 (1.17)	0.63	0.10
Oculomotor movement	0.60 (0.81)	0.63 (0.93)	0.90	0.027
Grading strength	1.61 (1.13)	1.49 (1.29)	0.64	0.10
Motor strength	1.10 (1.17)	0.96 (1.02)	0.55	0.13
Grading reflexes	1.28 (1.12)	1.08 (1.19)	0.42	0.17
Triceps reflex	0.69 (1.37)	0.98 (1.39)	0.34	0.21
Brachioradialis reflex	1.02 (1.24)	1.17 (1.47)	0.60	0.11
Ankle reflex	0.58 (1.11)	0.52 (1.38)	0.80	0.053
Composite neurologic examination confidence	7.62 (5.69)	7.66 (7.19)	0.97	0.0070

Note: Mean (SD). *p* value calculated using two-tailed, 2-sample equal variance *t* tests.

### Clerkship NE Evaluation Scores

Clerkship NE performance as evaluated by residents and faculty did not significantly differ between the LB (median = 4, interquartile range [IQR] = 3–4) and DI groups (median = 4, IQR = 3–4; *p* = 0.074, *r* = 0.070).

## Discussion

In this study, we evaluated the impact of a pitfall-oriented, DI didactic on medical student NE confidence and clerkship performance compared with an LB didactic. We found that this method was well received and rated by students to significantly improve their NE skills compared with the LB didactic. Contrary to our hypothesis, however, we did not find a significant difference in subjective NE confidence between groups, despite our study being sufficiently powered to detect that difference. We also did not find significant differences in clerkship physical examination performance between the LB and DI groups. These results highlight that while DI style didactics may be favored by students, LB and DI didactic formats may be comparable in their impact on student NE confidence and neurology clerkship physical examination performance. This is important because it suggests that either method could be used by educators in the clinical learning environment with similar impact on students' confidence and evaluations. However, DI may be preferred by educators because of students reporting greater perceived benefit from DI methods compared with LB didactics.

Similar to previous studies showing that greater physical examination confidence is associated with more clinical experience^25^ and completion of physical examination maneuvers,^26,27^ our study suggests that DI may improve self-reported medical student NE skills through observed practice in a classroom setting. Previous studies have demonstrated the benefit of using human examinees for learning examination skills,^28^ suggesting that practicing on other students may also prove to be beneficial for learning NE skills. Among the studies reviewed, several interventions used a single educational session^29-31^; however, the length of these sessions, the time from intervention to evaluation, and the methods varied significantly. Other studies also involved multiple sessions instead of a single session.^26,32-34^ These studies lead us to hypothesize that longitudinal DI didactics or DI didactics of longer duration may result in greater improvements in NE confidence and skills than those seen in our study.

Interesting discrepancies were observed in our study. For example, there was a discrepancy between self-reported NE confidence and self-reported efficacy of the DI didactic on clinical skills among clerkship students. This may have been due to our DI didactic preferentially improving skills over confidence. The teacher-centered method with DI allowed for paired-practice opportunities and gave students opportunities to improve skills; however, this alone may not have been enough to improve confidence. It is also possible that the positive effect of the DI didactic was masked by the clerkship itself because the pre-post assessments were 4 weeks apart and the clerkship would be expected to improve confidence regardless of didactic methods. Future studies could evaluate the impact of DI on student NE confidence immediately after a given didactic.

The lack of significant improvement in clerkship NE performance may also have multiple explanations. One contributor could be the hesitance of evaluators to assign scores lower than 3/5 because of a concern for how it may affect students' clerkship grades. Nearly all evaluation scores in our study fell within the 3/5–5/5 range, suggesting that evaluators at our institution may have used 3/5 as a frequent minimum score assigned to students. This bias would limit the ability of our study to detect significant changes in scores between groups. In addition, studies have shown that residents tend to give students higher evaluation scores than faculty,^35^ and more time with faculty tends to result in higher scores.^36^ The resident-to-faculty evaluation ratio between didactic groups, as well as time with evaluators, may have differed and may explain the lack of a significant clerkship performance improvement in the DI group. Future studies could evaluate the impact of this teaching method on clerkship performance using observed structured clinical examinations or other standardized resident or faculty evaluations to minimize potential bias and variability found in clerkship evaluations.

This study does have limitations. This DI didactic was implemented at a single center, which may limit its generalizability. Furthermore, to limit survey fatigue, we focused our evaluations on some, but not all, potential NE pitfalls. This may limit our ability to infer the effectiveness of the didactic on other examination skills, such as coordination and gait testing. While mental status is an important aspect of the NE, we excluded this aspect from our study to focus on more physical aspects of the examination that might benefit more from paired practice. Our evaluation of the didactic also did not include directly observed clinical assessments that could have bolstered the sensitivity for our study to detect significant changes in examination performance. The DI didactic also occurred later in the year than the LB didactic, which theoretically could have led to bias in measurements in NE confidence. This is unlikely to have affected our study, however, because all confidence scores, except for strength testing, were statistically similar before and after the clerkship regardless of academic block. In addition, the traditional didactic was taught by neurology clerkship directors while the DI didactic was taught by an MS4, which could have contributed to the lack of significant change in confidence between didactic methods. Although the literature suggests that peer teaching is noninferior to teaching by more experienced instructors,^37^ the perceived inferiority of learning from a student may also have been mitigated by faculty and residents directly overseeing peer learning.

Several important lessons were learned through the process of developing and implementing this intervention. First, cognitive load and behaviorist theories are valued by students in learning NE skills. Distilling the NE into high-yield relevant skills that students can focus on and giving them opportunities to practice allowed for greater engagement and learning than by a passive didactic alone. Second, when developing educational sessions for the NE, input from both learners and faculty is key. At our institution, we noted interesting differences in aspects of the NE that students and evaluators reported as most challenging for students. For example, the motor examination was more commonly noted as a challenge for students by evaluators than by students themselves. Other medical institutions will likely have unique areas of neurology that will most challenge their students, and our results suggest the need to survey all involved and tailor interventions to their learners. Third, student involvement in the development of such curricula offers valuable learning and career development experiences for trainees. Such projects create ample opportunities for students to learn more about neurology, develop valuable skills for residency and clinical practice, find mentors for clinical and academic pursuits, and contribute to education research.

Various methods could be used to promote sustainability of this intervention. Natural ways to sustain this DI didactic moving forward include involving future students who plan to apply to neurology residency, incorporating this educational initiative into the clinical educator track of the residency program at our institution, and involving the education chiefs of our institution's residency program. All these options would use preexisting programs and allow the novel didactic to continue without much further burden on clinical educators and staff.

Other institutions could implement this same method by performing similar surveys of students and RFF to evaluate student struggles with the examination. Clinical educators could then tailor these survey results and modify this DI didactic to their unique needs. Individuals interested in the educational materials for this novel didactic can contact the corresponding author (J.T.). With this study and DI didactic, we hope to increase discussions about and educational research on best practices for teaching the NE to learners during the neurology clerkship and beyond.

The development of high-quality, effective educational interventions in neurology has increased in importance and frequency in recent years.^38,39^ This is especially true for NE education because studies have demonstrated that learners consider neurology to be one of the most challenging specialties to learn.^40^ Much of the literature surrounding the development of neurologic and physical examination education has frequently focused on limited examination skills or novel teaching methodologies, with limited research exploring the use of more traditional, thorough NE skill didactics and workshops. This study contributes to the neurology education literature by suggesting that a DI didactic focused on common pitfalls, combined with paired practice, may enhance medical students' NE skills during the neurology clerkship. Educational interventions with a cognitive load and behaviorist conceptual framework seem to be particularly beneficial in learning these skills. More research is needed to define how to best implement this framework in the neurology clerkship, develop better assessment mechanisms to detect meaningful improvements in student NE confidence, and develop and determine the most effective methods for teaching the NE to learners.

## Glossary

DI: direct instruction
LB: lecture-based
MS3: third-year medical student
NE: neurologic examination
RFF: residents, fellows, and faculty
UTHSA: University of Texas Health San Antonio
